# Cumberland Ankle Instability Tool (CAIT) demonstrates comparable predictive validity to Foot and Ankle Ability Measure (FAAM) for return to sport after anatomic ankle ligament reconstruction

**DOI:** 10.1002/jeo2.70480

**Published:** 2025-10-28

**Authors:** Ibrahim Saliba, Olivier Grimaud, Vincent Fontanier, Frederic Khiami, Yoann Bohu, Nicolas Lefevre, Alexandre Hardy

**Affiliations:** ^1^ Orthopedic Surgery Department Cochin hospital Paris France; ^2^ Orthopedic Surgery Department Clinique Du Sport Paris France; ^3^ Medinetic Learning Paris France

**Keywords:** anatomic ankle lateral ligament reconstruction, CAIT, chronic lateral ankle instability, FAAM, Return to Sport (RTS)

## Abstract

**Purpose:**

Anatomic ankle ligament reconstruction (AALR) has shown positive outcomes in managing chronic lateral ankle instability (CLAI). Patient‐reported outcome measures (PROMs) are essential for evaluating treatment efficacy, but the optimal tool for this population remains unclear. This study aimed to compare the discriminative capacity and predictive ability for Return to Sport (RTS) between two validated PROMs: the Foot and Ankle Ability Measure (FAAM) and the Cumberland Ankle Instability Tool (CAIT), in patients undergoing AALR.

**Methods:**

A retrospective study included 140 patients who underwent arthroscopically‐assisted AALR with gracilis autograft or allograft between January 2019 and January 2023, with a minimum 12‐month follow‐up. Patients completed FAAM and CAIT questionnaires preoperatively and at 3, 6 and 12 months postoperatively. Correlations between FAAM and CAIT scores were assessed using Spearman coefficients across different timelines. Discriminative capacity and predictive ability for RTS were also compared using Wilcoxon test, ROC and Area under the Curve (AUC) analysis.

**Results:**

Strong correlations between CAIT and FAAM were observed preoperatively (*r* = 0.53) and postoperatively at 3 months (*r* = 0.56), 6 months (*r* = 0.64) and 12 months (*r* = 0.69). Both scores discriminated significantly between RTS and non‐RTS groups, with higher scores in the RTS group at 3 months (CAIT: 23 vs. 17, *p* < 0.001; FAAM: 84 vs. 72, *p* < 0.001), 6 months (CAIT: 27 vs. 19, *p* < 0.001; FAAM: 90 vs. 74, *p* < 0.001), and 1 year (CAIT: 29 vs. 24, *p* < 0.001; FAAM: 93 vs. 84, *p *< 0.001). AUC analysis revealed no significant difference in predictive ability for RTS between CAIT and FAAM at 3 months (n.s.), 6 months (n.s.) and 12 months (n.s.).

**Conclusion:**

Both CAIT and FAAM demonstrated comparable discriminative capacity and predictive ability for Return to Sport following AALR; given its brevity, ease of administration, and specificity to ankle instability, CAIT is a valid and efficient alternative to FAAM for postoperative outcome assessment in this population.

**Level of Evidence:**

Level III.

**Abbreviations**
AALR: anatomic ankle ligament reconstruction
ADL: activities of daily living
ATFL: anterior talo‐fibular ligament
AUC: area under the curve
CAIT: cumberland ankle instability tool
CFL: calcaneo‐fibular ligament
CLAI: chronic lateral ankle instability
FAAM: foot and ankle ability measure
LEFS: Lower Extremity Function Scale
MRI: magnetic resonance imaging
n.s.: nonsignificant
PROM: patient‐reported outcome measure
r: Spearman correlation coefficient
ROC: receiver operating characteristic
RTS: return to sport
SMS: short message service

## INTRODUCTION

The incorporation of patient‐reported outcome measures (PROMs) within the assessment and treatment process is vital for delivering patient‐centred care and evaluating treatment effectiveness from the patient's perspective [13]. In recent years, the significance of PROM data has been increasingly recognized [11, 26]. Among the commonly employed PROMs in studies investigating effective treatment strategies for chronic lateral ankle instability (CLAI) is the Foot and Ankle Ability Measure (FAAM) [13]. However, it has been identified that certain items within the FAAM, such as basic Activities of daily living ADLs that don't stress ankle stability (e.g., ‘cooking’, ‘cleaning’, ‘shopping’ ‘climbing low stairs’) and tasks in nonchallenging environments, which don't reflect the functional deficits seen in CLAI, may not be relevant to CLAI patients [14]. The Cumberland Ankle Instability Tool (CAIT) has demonstrated acceptable construct validity, internal reliability, and test‐retest reliability, effectively distinguishing between individuals with and without CLAI [12, 13]. Furthermore, it is a brief, CLAI‐specific and a widely accepted instrument endorsed by the International Ankle Consortium for measuring symptoms and impairments associated with CLAI [17]. Although CAIT has not yet been formally validated as a postoperative outcome measure, this study explores its discriminative and predictive value in patients undergoing AALR.

Despite the widespread use of both FAAM and CAIT, the relationship between these instruments in patients undergoing Anatomic Ankle Ligament Reconstruction (AALR) has not been thoroughly investigated [12, 13, 27]. Existing literature provides limited evidence regarding the correlation between FAAM and CAIT scores, particularly in the context of postoperative recovery and Return to Sport (RTS) [19, 27]. Additionally, there is a lack of studies directly comparing the discriminant validity and predictive ability of these tools for RTS assessment [12, 19, 27].

The objective of this study was to investigate the correlation between FAAM and CAIT scores in CLAI patients undergoing anatomic reconstruction of lateral ankle ligaments, as well as to compare the discriminant validity and predictive ability for RTS between these two instruments in this specific population.

It is hypothesized that the CAIT score possesses measurement properties comparable to those of the FAAM as a PROM for assessing RTS in patients treated for CLAI, with the added advantage of being shorter, less time‐consuming and more specific to ankle instability—features that may enhance clinical efficiency by reducing completion time and providing ankle‐specific monitoring.

## MATERIAL AND METHODS

This study was approved by the Institutional Review Board (COS‐RGDS‐2025‐07‐005‐HARDY‐A). Informed consent was obtained from all the patients.

### Study design and inclusions

This retrospective cohort study encompassed patients >18 years old with CLAI who underwent AALR at a sports surgery centre between January 2019 and January 2023. The centre's database was utilized to identify patients based on relevant diagnosis and procedure codes. The minimum follow‐up period was set at 12 months.

Exclusion criteria: Patients who had a history of lower extremity surgery in the last 2 years preceding AALR procedure, as well as patients with associated talar or tibial cartilage lesions measuring >10 mm in maximum diameter, regardless of lesion grade, were excluded. All included cartilage lesions (grades 2–4) were <10 mm and therefore considered nonsignificant as recent literature [1, 5, 22] showed that lateral small cartilaginous lesions do not affect clinical outcomes postoperatively. One patient with a chronic grade 4 lesion required a concomitant cartilage procedure, and therefore was excluded from the statistical analysis (see Table 1).

**Table 1:** This table provides a summary of the demographic data and descriptive characteristics of this study population.

Description of patients with ligament reconstruction	Overall (*N* = 140)
Age, median (25%–75%)	29 (24–39)
Sex, *n* (%)	
Female	66 (47.1)
Male	74 (52.9)
Operated ankle, *n* (%)	
Right	75 (64.1)
Left	42 (35.9)
Delay to Return to Sport, Median (25%–75%)	992 (315–2398)
**Global** Stage as per arthroscopic classification of ATFL, *n* (%)	
Stage 0: Normal and continuous LTFA	3 (2.8)
Stage 1: Stretched LTFA without tear	2 (1.9)
Stage 2: LTFA detachment	42 (38.9)
Stage 3: Thin ligament, with or without scar tissue	27 (25.0)
Stage 4: Scar tissue without residual ligament	33 (30.6)
**Global** Grade of cartilage status, n (%)	
Grade 0: Normal	70 (68.0)
Grade 1: Nearly normal (Softening and/or superficial cracks)	18 (17.5)
Grade 2: Abnormal <50% thickness	7 (6.8)
Grade 3: Abnormal (Does not reach calcified layer, reaches calcified layer, up to subchondral bone)	5 (4.9)
Grade 4: Severely abnormal (Affects the entire cartilage thickness with extension to subchondral bone)	1 (1 0)^a^
Sport practice, *n* (%)	
Competition	49(38.0)
Occasional leisure (active)	22(17.1)
Regular leisure	37(28.7)
Professional	18(14.0)
Sedentary	3 (2.3)
Type of Sport, *n* (%)	
No sport	10(7.8)
Linear sport (Jogging, cycling)	31(24.0)
Pivot sport (Skiing, tennis, squash, badminton, volleyball, dance, gymnastics, golf)	35 (27 1)
Pivot contact sport (Football, rugby, basketball, handball, hockey, combat sports)	53 (41.1)

^a^ Patient excluded from data analysis.

### Indication for surgery

Criteria for diagnosing CLAI included a history of recurrent ankle sprains, ankle pain or swelling, a sensation of instability, or symptoms limiting daily or sports activities [8, 24].

Physical examination assessed objective laxity through a comparative evaluation of both ankles using the anterior drawer test, forced varus stress, and rotational stress [20]. The presence of a sulcus sign during forced varus was considered indicative of ligamentous laxity [20].

Standard imaging included anteroposterior and lateral weight‐bearing radiographs. The integrity of the Anterior Talo‐Fibular Ligament (ATFL) and Calcaneo‐Fibular Ligament (CFL) was evaluated using Magnetic Resonance Imaging (MRI). Anatomic reconstruction, rather than direct repair, was performed when the ATFL was absent or markedly thinned, a finding confirmed intraoperatively [20].

### Surgical technique

All patients underwent the same surgical technique performed by three surgeons, which involved direct reconstruction of the ATFL and CFL using the gracilis tendon [21]. The autograft was harvested from the ipsilateral knee. Gracilis allografts were used for three patients whose ipsilateral hamstring tendons had already been harvested for reconstruction of other joint ligaments. Under arthroscopic guidance, the graft was fixed distally in a talar tunnel for ATFL reconstruction and in a calcaneal tunnel for CFL reconstruction. Proximal suspension to the fibula was achieved by passing the graft through a fibular tunnel.

### Postoperative protocol

Postoperatively, patients were permitted immediate weight‐bearing while wearing a walking boot for a duration of 3 weeks. A physiotherapy protocol was prescribed to facilitate the strengthening of the ankle muscles, as well as to regain balance and proprioception. RTS to preinjury level was allowed for patients with an Ankle GO score >14 [10] based on the physiotherapist or physician assessment. Ankle‐GO, a validated functional test combining strength, balance, and self‐reported metrics, was used to guide RTS decisions; [10] however, as it is not a PROM, the focus of this study was placed on whether CAIT and FAAM—two PROMs—could independently reflect functional recovery and predict RTS outcomes.

### Outcome measures and variables

The primary outcome measures was the correlation between the FAAM and CAIT scores. An analysis of the discriminant validity and the predictive capacity to the ability to RTS after AALR was also carried out. The FAAM consists of 29 items distributed across two subscales, namely FAAM‐Sport and FAAM‐Activities of Daily Living. Each item is scored between 0 and 4 with lower scores reflecting less favourable outcomes [23]. The Cumberland Ankle Instability Tool (CAIT) is a 9‐item instrument specifically designed to assess self‐reported impairments associated with CLAI [12]. The CAIT employs a scoring system ranging from 0 to 30, where lower scores indicate greater severity of CLAI‐related symptoms [12].

The secondary outcome measure was the rate of return to sport (RTS) at the preinjury level following surgery. Postoperative follow‐up was conducted through clinical visits with the surgeon at 3 months, 6 months, and 1 year. During these visits, patients were assessed using the Ankle‐GO test, which was also performed when applicable by physiotherapists or rehabilitation centre physicians. RTS was authorized when the Ankle‐GO score exceeded 14, in line with validated thresholds. In addition, a follow‐up questionnaire—sent via mail or SMS and regularly re‐sent to non‐responders—collected patient‐reported outcomes including FAAM, CAIT, a binary (Yes/No) response confirming whether they had returned to sport, and the timing of their actual RTS. While Ankle‐GO served as the clinical criterion to authorize RTS, the self‐reported Yes/No RTS response was used to confirm that the patient had effectively resumed sport activity and to document when this occurred. Delay to return to sports (RTS‐delay) for each participant was defined as the time interval between the date of surgery and the first full training session or competition performed at the preinjury level was recorded. RTS‐delay is expressed in weeks, and because its distribution was skewed, it is reported as the median with inter‐quartile range (25th–75th percentiles) in Table 1.

Patients who did not practice sports prior to surgery (*n* = 10) were excluded from all analyses related to RTS, including discriminant and predictive validity analyses. However, these patients were included in the analysis of FAAM and CAIT scores, as both instruments assess broader aspects of ankle function and stability beyond sport‐specific activities.

### Data collection

Global stage of ATFL lesion was determined per‐operatively as per the arthroscopic classification of chronic ATFL rupture in CLAI described by Thès et al. [30]. Regular activity was defined as at least once a week, whereas occasional practice was defined as less than once a week [33].

### Participants and sample size

A total of 140 patients were included in the study, consisting of 74 males and 66 females. The median age of the participants was 29 (24–39) years. Table 1 provides a summary of the demographic data and descriptive characteristics of the study population.

### Statistical analysis

Statistical analyses were conducted using R software, version 3.5.0, which can be accessed at the URL https://www.R-project.org.

No a priori sample size calculation was performed. Instead, a post hoc precision analysis was conducted for Spearman's rank correlation coefficient. With a correlation estimate greater than 0.50, the sample size of 140 patients provided a two‐sided 95% confidence interval with a width of approximately 0.33.

Descriptive statistics were used to summarize continuous quantitative variables, with means and interquartile ranges reported unless otherwise specified. Dichotomous variables were described in terms of the number of events and their percentages. All questionnaire scores were reported on a scale of 0 to 100, where 0 represented very poor and 100 denoted excellent.

The correlation between the CAIT score and the FAAM score was assessed using the Spearman correlation coefficient. The strength of correlation was categorized as ‘strong’ (*r* > 0.5), ‘moderate’ (0.5 < *r* < 0.3), or ‘weak’ (0.3 < *r* < 0.1).

To assess the discriminant validity of each score for RTS, Wilcoxon test was utilized to compare the medians of CAIT and FAAM at 3 months and 6 months in patients who resumed their sports or not following surgery. A *p* < 0.05 was deemed statistically significant. Moreover, Receiver Operating Characteristic (ROC) curve analysis was employed, providing the Area Under the Curve (AUC). Higher AUC values indicate greater discriminant validity, with values exceeding 0.6 reflecting increasing levels of discrimination [15].

To evaluate predictive ability, DeLong's test for two correlated ROC curves [3] was employed to compare the predictive capacity of both CAIT and FAAM scores.

## RESULTS

### Correlation between CAIT and FAAM

**Figure 1:**
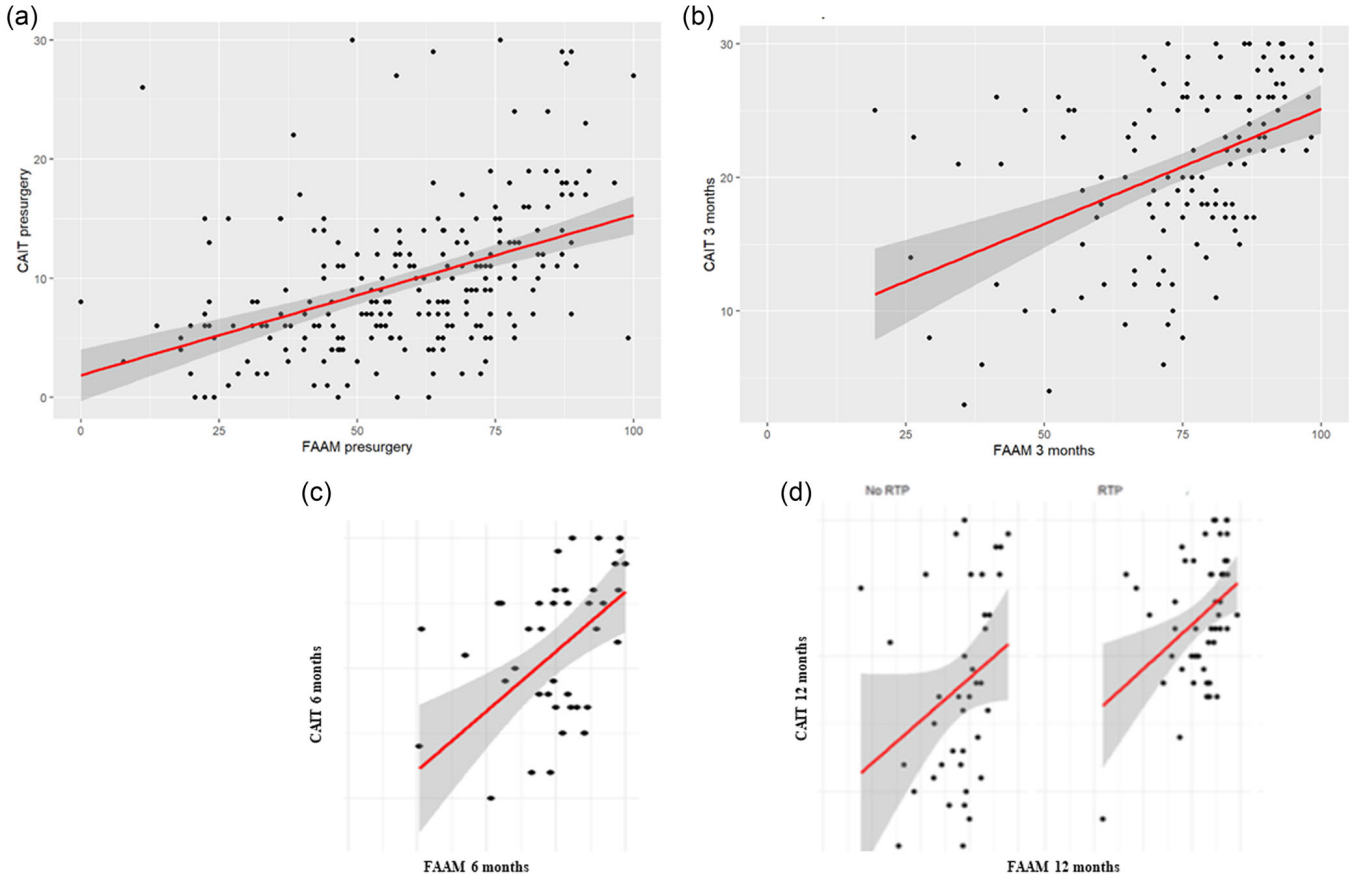
Correlation between CAIT and FAAM scores at different time points. (a) Preoperative correlation (*r* = 0.53). (b) Correlation at 3 months postoperatively (*r* = 0.56). (c) Correlation at 6 months postoperatively (*r* = 0.64). (d) Correlation at 12 months postoperatively; *r* = 0.68 in patients who returned to sport (RTS) and *r* = 0.65 in those who did not. Overall correlation across all time points: *r* = 0.69. These results demonstrate a progressively stronger correlation between CAIT and FAAM scores during follow‐up. CAIT, Cumberland Ankle Instability Tool; FAAM, foot and ankle ability measure.

The CAIT demonstrated a strong (*r* > 0.50) and significant correlation with the FAAM before surgery (*r* = 0.53) and at 3 months (*r* = 0.56), 6 months (*r* = 0.64) and 12 months (*r* = 0.69) postsurgery (Figure 1). Additionally, a strong correlation was observed between the two scores within both patient groups—RTS and no RTS—at 12 months, with *r* = 0.68 for the RTS group and *r* = 0.65 for the no RTS group (Figure 1).

### Discriminant validity

Both CAIT and FAAM scores showed strong discriminant validity, being significantly higher in the RTS group compared to individuals who no longer participated in their primary preinjury sport. In the RTS group, the median CAIT score was 23 [20, 21, 22, 23, 24, 25, 26, 27] at 3 months, 27 [25, 26, 27, 28, 29, 30] at 6 months and 29 [27, 28, 29, 30] at 1 year, whereas it was 17 [12, 13, 14, 15, 16, 17, 18, 19, 20, 21, 22, 23, 24] (*p* < 0.0011), 19 [13, 14, 15, 16, 17, 18, 19, 20, 21, 22, 23, 24, 25] (*p* < 0.0011) and 24 [18, 19, 20, 21, 22, 23, 24, 25, 26, 27, 28] (*p* < 0.0011) in patients who did not resume sports at 3 months, 6 months and 1 year, respectively (Table 2). Similarly, the median FAAM score in the RTS group was 84 [76–90] at 3 months, 90 [83–93] at 6 months and 93 [89–97] at 1 year, while it was 72 [60–81] (*p* < 0.001), 74 [58–85] (*p* < 0.001) and 84 [63–94] (*p* < 0.001) in patients who did not return to sports at 3 months, 6 months and 1 year, respectively.

**Table 2:** Showing the evolution of CAIT score from preoperative phase to 1 year following surgery in both RTS and No RTS groups.

Time	No RTS *n* = 54	RTS *n* = 86	Overall *N* = 140	*p*‐Value
Preoperative CAIT	8 (6–11)	8 (6–12)	8 (6–11)	n.s.
3 months post‐op CAIT	17 (12–24)	23 (20–27)	22 (17–26)	<0.0011*
6 months post‐op CAIT	19 (15–25)	27 (25–30)	26 (20–29)	<0.0011*
1 year post‐op CAIT	24 (18–28)	29 (27–30)	28 (21–30)	<0.0011*

Abbreviations: CAIT, Cumberland Ankle Instability Tool; RTS, Return to Sport.

*p < 0.05.

**Figure 2:**
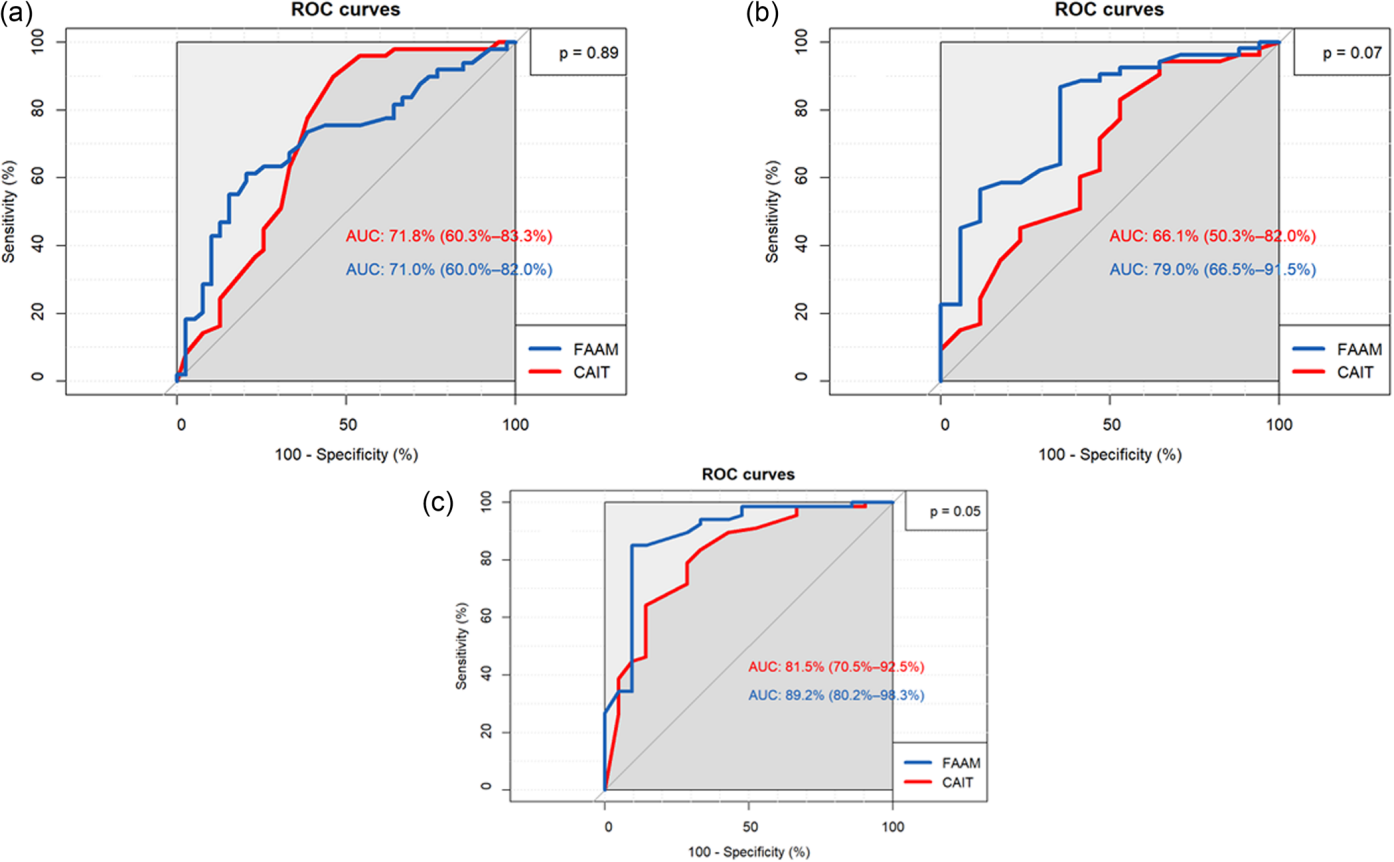
Receiver Operating Characteristic (ROC) curves comparing the predictive ability of CAIT and FAAM scores for return to sport (RTS) at different postoperative time points. (a) At 3 months postoperatively. (b) At 6 months postoperatively. (c) At 12 months postoperatively. Area under the curve (AUC) values are shown for each measure, indicating comparable discriminative performance of CAIT and FAAM in identifying patients who successfully returned to sport. CAIT, Cumberland Ankle Instability Tool; FAAM, foot and ankle ability measure.

Moreover, as shown in Figure 2, both CAIT and FAAM scores showed AUC values >0.6 (or >60%) at 3, 6 and 12 months following surgery.

### Predictive capacity of the ability to RTS

CAIT showed an AUC of 0.7 (71.8%), 0.66 (66%) and 0.82 (81.5%) at 3, 6 and 12 months, respectively. FAAM also had an AUC of 0.7 (70%), 0.79 (79%) and 0.89 (89.2%) at 3, 6 and 12 months, respectively. Comparison of the ROC curves for the two scores revealed no significant differences at 3 months (n.s.), 6 months (n.s.) and 12 months (n.s.) (Figure 2).

### Return to sport

Forty‐eight patients out of 140 (34%) returned to sports at 3 months following surgery. At 6 months, 86 patients (61%) returned to sports. At 1 year, 102 patients (73%) resumed their sporting activities.

## DISCUSSION

The main findings of this study showed that CAIT score which has been already validated in CLAI population could be a relevant instrument to measure patients reported outcomes following the anatomic reconstruction of lateral ankle ligaments in CLAI patients [13, 31]. CAIT showed a strong correlation with FAAM with a good discriminant validity between RTS and no‐RTS groups. Furthermore, there was no statistical difference between FAAM and CAIT regarding the predictive ability to assess RTS at 3 months and 6 months following surgery. These results indicate that CAIT demonstrates comparable measurement properties to FAAM, with the added advantages of brevity, ankle‐specificity and established cutoff values. Thus, CAIT may be preferred in clinical practice. It should be noted that 3 months may represent an early time point for evaluating RTS, as only a minority of patients had resumed sport activity by then. RTS assessments at 6 and 12 months are likely to provide a more reliable reflection of functional recovery.

Hansen et al. [9] considered that FAAM is the best compromise to be used in ankle instability patients. In fact, after analyzing 17 identified questionnaires used in ankle instability population, they identified only 3 scales as having some degree of patient involvement: FAAM, CAIT and Lower Extremity Function Scale (LEFS) [9]. Although they concluded that no existing PROM is completely adequate to assess ankle instability, they considered FAAM as the best choice based on psychometric validation methods [9].

In fact, FAAM is among the most utilized PROMs to assess the efficacy of treatment management in patients with ankle instability [4, 16, 28, 29]. However, FAAM includes 29 items and it typically requires approximately 5–10 min to complete [23], whereas the CAIT, consisting of only 9 items, can be completed in 2–3 min [2, 12]. Moreover, as it was previously mentioned, FAAM includes several items which are not relevant to patients with CLAI because it is not a specific score to ankle instability [14]. Some authors developed recently a quick‐FAAM in an attempt to address the aforementioned disadvantages of FAAM and to increase its relevance to CLAI patients [14]. They retained 7 items from the Sport Subscale and 5 items from the Activities of Daily Living (ADL) subscale making it a 12‐item single scale [14]. In contrast, CAIT is a concise 9‐item scale, specifically designed for ankle instability assessment, and filled out for both the right and left ankles, thus minimizing patient burden, increasing reliability and assessing both ankles individually [12, 31]. It also has been validated in different languages: English, Brazilian‐Portuguese, Spanish, Korean, Chinese, French and Dutch [2, 6, 12, 18, 25, 31, 32]. Another advantage of CAIT is that it has a cutoff value for instability which is actually set at 24 by the International Ankle Consortium (IAC) allowing physicians to have a universal reference point with good sensibility and specificity [7]. Scores below 24 indicate unstable ankles while scores above 24 indicate stable ankles. On the other hand, the recommended cutoff values by the IAC for the FAAM scale (FAAM‐ADL < 90 and FAAM‐Sport <80) [7] did not seem to be as accurate as CAIT cutoff. In their study including 211 participants, Li et al. [19] showed that CAIT had a sensibility of 80.7% and specificity of 84.9% when setting the cutoff at 24, while 97% of the participants with chronic ankle instability, especially those with mild functional impairment, were excluded when FAAM was used with the abovementioned cutoff values. All the above‐mentioned findings support the CAIT score as a viable alternative to the FAAM for assessing postoperative outcomes in patients undergoing AALR surgery.

The main limitation of this study is its retrospective design, which may introduce potential selection bias. Efforts were made to mitigate this bias by minimizing loss to follow‐up and facilitating patient engagement through clear communication and regular reminders. While the inclusion of motivated and easily traceable patients may introduce some bias, it also ensured higher response rates and more complete data, which are essential in PROM‐based studies. Questionnaires were designed to be user‐friendly, and patient interest was maintained by emphasizing the relevance of the study to their ongoing care. Automated reminders were sent via SMS and email before each follow‐up visit, and incomplete questionnaires were completed during in‐person visits whenever possible. Another limitation is that 14 patients with grade 2–4 cartilage lesions <10 mm in diameter were included. This may be a confounding effect on the outcomes. However, recent evidence [1, 5, 22] suggests that small osteochondral lesions of the talus (<10 mm diameter), even with increased depth or higher grade, do not significantly affect clinical outcomes after lateral ankle ligament reconstruction and are unlikely to confound PROM results. Furthermore, the only patient with cartilage lesion who underwent concomitant cartilage procedure was excluded from statistical analysis. An additional limitation of the present study is the duration of follow‐up, which was limited to 12 months. Although longer follow‐up would have been valuable, consistent data were only available up to 12 months for the entire cohort. Extending the analysis to 2 years would have substantially reduced the sample size due to loss to follow‐up.

This study is clinically relevant. This is the first direct comparison of FAAM and CAIT in AALR patients, showing CAIT as a reliable and efficient alternative for monitoring recovery and guiding RTS decisions. It offers practical guidance for clinicians seeking efficient, ankle‐specific tools to support postoperative decision‐making and outcome monitoring.

## CONCLUSION

In conclusion, CAIT is an effective PROM for evaluating outcomes after AALR in patients with CLAI, showing strong correlation and comparable predictive power to FAAM. Given its brevity, ease of use, and specificity to ankle instability, CAIT should be preferred in routine clinical practice where efficiency and targeted assessment are key.

## AUTHOR CONTRIBUTIONS

Ibrahim Saliba contributed to the study design and article writing. Olivier Grimaud participated in data collection. Vincent Fontanier participated in data analysis. Frederic Khiami participated in data collection and provided critical feedback. Yoann Bohu participated in data collection and provided critical feedback. Nicolas Lefevre provided critical feedback. Alexandre Hardy contributed to the study design, article writing and critical feedback.

## CONFLICT OF INTEREST STATEMENT

The authors declare no conflict of interest.

## ETHICS STATEMENT

COS‐RGDS‐2025‐07‐005‐HARDY‐A. Informed consent was obtained from all the patients.

## Data Availability

The data that supports the findings of this study are available in the supplementary material of this article.
